# Accelerating genomic data publishing and sharing

**DOI:** 10.1016/j.gdata.2013.05.001

**Published:** 2013-05-28

**Authors:** Jian-Bing Fan, John Quackenbush, Bart Wacek

**Affiliations:** a Illumina, Inc., San Diego, CA, USA; b Dana-Farber Cancer Institute, Boston, MA, USA; c Elsevier Publishing, Boston, MA, USA

Elsevier Publishing is pleased to announce the launch of *Genomics Data*, an open access journal that considers articles on all aspects of genome-scale analysis.

Over the past 20 years, genomics technology evolutions have drastically transformed biomedical researches. Powerful genomics tools have enabled whole-genome gene expression analysis, genotyping of millions of genetic markers in hundreds and thousands of individuals, and ultimately the complete genome sequencing of many organisms. Large amounts of genomics datasets have been generated and are being generated routinely. We see a huge need to publish genomics research results quickly and to allow the research community to openly share and exchange data. Toward this goal, *Genomics Data* aims to provide a useful format that links authored content with structured standard information about a genomic or functional genomic study, which shall maximize data searching, mining and reporting.

*Genomics Data* publishes high quality and standardized reports summarizing results of microarray or sequencing studies in all types of organisms. The genomic and functional genomic data/results are intended to serve as points of record that are enriched with data generation strategies and methods, data QC metrics, data analysis process and algorithms, biological interpretation and conclusions. All articles will be peer-reviewed and verified by the editorial team.

Authors may construct their reports with a downloadable genome report document template with the following or part of the following information: (1) Title of the paper; (2) Author name and affiliations; (3) Key words; (4) Platforms: microarray and/or next-gen sequencing (NGS), for example, genomic sequencing, RNA-Seq, Epi-Seq (e.g. Methylation, ChIP-Seq). The text can be organized into (1) Abstract; (2) Introduction; (3) Study Design; (4) Methods, SOP/protocol; (5) Results: i) raw data, to be submitted to GenBank, NCBI/NIH (SRA, GEO), and submission numbers should be cited in the manuscript; ii) data format, e.g. XML; iii) results (figures, tables); iv) data description and interpretation; (6) Conclusion and Discussion.

In addition to original research articles, authors may also consider making contributions in the forms of (1) review, (2) method and/or SOP, (3) meeting report, and (4) commentary and perspective articles.

Standard operating procedures (SOPs) should follow a general format of title, authors, institutions, overview and procedures used, and should categorize the type(s) of processes involved, revision versions, dates and any underlying dependencies. *Genomics Data* encourages submission of details such as command-line arguments or other run-time parameters, and operational thresholds. The sequential procedure of computational and manual operations for data capture and calculation should describe but not be limited to the following: i) assumptions involved; ii) steps for reproducibility; iii) points at which the quality of the process and its output may be evaluated.

*Genomics Data* is user friendly, offering creative apps that allow authors to bring their data into the publishing environment. One example of this is a *Genomics Data* app that resides in the cloud. When the data sets are stored in the cloud, a simple to use app can help authors take the data into the review/publishing environment and thus add the credibility that can come only from the formal peer review process. This credibility is further enhanced by expert reviewers that have the additional means to review your work while using the same analytic tools that you used in the cloud. Data deposits and wide dissemination complete the user friendly functionality of the app. We intend to make articles and data published in *Genomics Data* indexed and searchable in major public databases such as PubMed Central.

